# CLAPO syndrome: A case report emphasizing diagnosis and treatment options

**DOI:** 10.1016/j.jdcr.2025.03.015

**Published:** 2025-04-01

**Authors:** Noah Hewitt, Josiah Williams, Zachary Zinn

**Affiliations:** a Department of Medicine, West Virginia University School of Medicine, Morgantown, West Virginia; b Department of Dermatology, West Virginia University School of Medicine, Morgantown, West Virginia

**Keywords:** capillary malformation, case report, CLAPO, hemangioma, pik3ca, port wine stain

## Introduction

Capillary malformation of the lower lip, Lymphatic malformation of the face and neck, Asymmetry of face and limbs, Partial/generalized Overgrowth (ie CLAPO syndrome) is a phenotype of the PIK3CA-related overgrowth spectrum (PROS) that typically presents with a distinct midline lower lip capillary malformation.^1^ The lower lip capillary malformation can mimic an infantile hemangioma at birth causing diagnostic confusion.

## Case report

**Fig 1:**
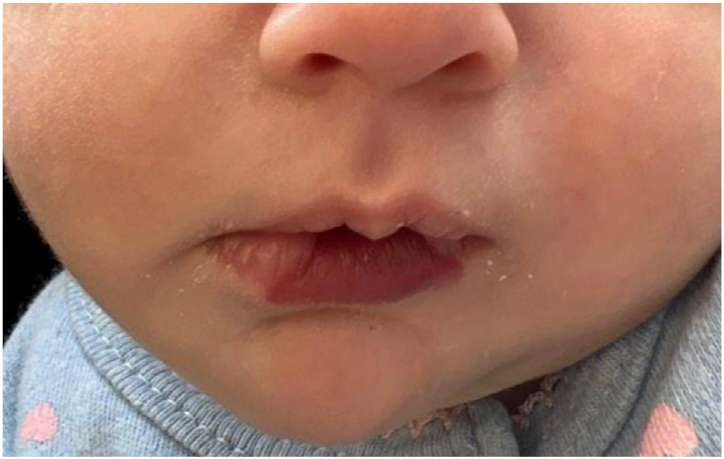
Symmetric, well-demarcated *purple* patch of the lower lip.

A 5-week-old girl presented to clinic for a midline purpuric patch of the lower mucosal lip involving the vermilion border (Fig 1). The lesion was fully formed at birth and had not grown or otherwise changed. History was notable for fraternal twin pregnancy and delivery at 35 weeks due to pre-eclampsia. The patient was started on topical timolol for suspected infantile hemangioma with plans for transition to propranolol with any growth or change. Unfortunately, the patient was lost to follow-up until 7 months of age. At that time, the patient presented to the emergency department for nontender neck swelling. An ultrasound revealed a cystic, hypoechoic structure suspicious for a venous or lymphatic malformation. At follow-up in dermatology clinic at 8 months of age, the lower lip lesion remained largely unchanged. The neck swelling was palpable, but no overgrowth was noted. Magnetic resonance imaging with contrast revealed multiple enhancing lesions consistent with venous or lymphatic malformations of the neck and central face, including the lower lip, floor of the mouth, and bilateral submandibular spaces, as well as T2 enhancement of the lip. The patient was thus diagnosed with CLAPO syndrome, a phenotype of PROS, based on the characteristic lower lip capillary malformation and associated vascular malformations of the head and neck. The patient was referred to vascular anomalies multidisciplinary clinic. She was also referred to otolaryngology for the facial and neck vascular malformations, who concluded that the vascular malformations were not at risk of compromising her airway.

At vascular anomalies multidisciplinary clinic, medical therapy with alpelisib or sirolimus were considered the most appropriate treatment options since they are beneficial in PROS and since procedural intervention such as excision or sclerotherapy would be high risk and unlikely to be effective given the multifocal nature of the patient’s vascular malformations. However, since her vascular malformations were small and there was no apparent overgrowth, the patient’s parents declined treatment. Definitive genetic testing was also deferred. The patient follows up for close monitoring and periodic imaging with plans to start alpelisib if the vascular malformations enlarge or overgrowth is noted.

## Discussion

First described in 2008, CLAPO syndrome is an acronym that stands for its clinical features of capillary malformation of the lower lip, lymphatic malformation of the face and neck, and asymmetry with partial or generalized overgrowth.^1^ Most cases arise from a somatic gain-of-function mutation in PIK3CA, making it a phenotype of the PROS.^2^

PROS comprises a variety of tissue overgrowth syndromes that commonly present with vascular malformations.^2^^,^^3^ Overgrowth of other tissues may also develop, including brain, limbs, digits, trunk, and face.^3^^,^^4^ Overgrowth may be congenital or may develop during childhood.^3^ The varied clinical phenotypes are attributed to a somatic mutation which may affect a myriad of tissues depending on when and where the mutation occurs during development.^2^ Multiple PROS phenotypes have been described in addition to CLAPO syndrome, including Klippel-Trenaunay syndrome, characterized by extremity venous malformations, capillary malformations, and limb overgrowth.^3^

Like other PROS phenotypes, somatic mosaicism largely explains the variable expressivity seen in CLAPO syndrome, with cases ranging from a lip capillary malformation without significant lymphatic malformations to asymmetric overgrowth and bony hypertrophy.^2^ The lower lip capillary malformation is present in all cases.^2^ This is often the presenting feature and can immediately raise suspicion for CLAPO syndrome since it is characteristically on the midline lower lip, often crossing the vermilion border or extending into the intraoral mucosa.^3^ Since this capillary malformation can mimic an infantile hemangioma, a thorough history is helpful. Growth in the first weeks of life suggests infantile hemangioma, while a lesion stable since birth is more suggestive of capillary malformation. In the first few months of life, a follow-up visit in 1 or 2 months can also help make the diagnosis since infantile hemangiomas typically grow in the first 6 months of life while capillary malformations do not.^2^ Venous and lymphatic malformations typically affect the cervicofacial region and tongue but may also occur on the extremities in the minority of cases, supporting a routine full-body skin exam at diagnosis and follow-up.^2^ Tongue lesions warrant close monitoring since they tend to grow and may lead to hemorrhage.^2^ When present, asymmetry and overgrowth may be subtle and may develop over time; therefore, the apparent lack thereof should not exclude the diagnosis.^2^ Risk of progressive overgrowth and morbidity associated with extensive vascular malformations warrant regular follow-up for monitoring. Although there are no comprehensive imaging guidelines for CLAPO, a baseline magnetic resonance imaging could be considered to characterize vascular malformations.^5^ Repeat imaging of the head and neck could also be considered when there is clinical evidence of somatic overgrowth near vital structures.

Historically, the treatment for PROS has been aimed at symptom reduction.^4^ Pulsed dye laser can treat the capillary malformations on the lower lip to improve cosmetic outcome.^6^ Since capillary malformations may become thickened with age, there may be a functional benefit as well.^2^ Oral sirolimus is widely used for the treatment of PROS, which confers benefit by inhibiting mammalian target of rapamycin, a downstream regulator of the PIK3CA pathway.^4^^,^^7^ Sirolimus can reduce the size of lymphatic malformations in CLAPO syndrome and may confer benefit in progressive overgrowth.^8^ Alpelisib is another systemic option. Alpelisib is an oral PIK3 inhibitor originally developed for the treatment of PIK3CA-mutated breast cancer.^4^^,^^9^^,^^10^ Recently, alpelisib was approved by the U.S. Food and Drug Administration for treatment of PROS in patients 2 years of age or older.^4^ It is a targeted therapy that works by slowing the progression of overgrowth, often meaningfully reducing overgrowth by 6 months of treatment.^4^^,^^10^ While head-to-head trials between oral alpelisib and sirolimus are lacking, alpelisib is efficacious in PROS, making it a promising new targeted therapy.^4^

## Conflicts of interest

None disclosed.
